# Biallelic variants in *CEP164* cause a motile ciliopathy‐like syndrome

**DOI:** 10.1111/cge.14251

**Published:** 2022-11-03

**Authors:** Laura A. Devlin, Janice Coles, Claire L. Jackson, Miguel Barroso‐Gil, Ben Green, Woolf T. Walker, N. Simon Thomas, James Thompson, Simon A. Rock, Ruxandra Neatu, Laura Powell, Elisa Molinari, Genomics England Research Consortium, Ian J. Wilson, Heather J. Cordell, Eric Olinger, Colin G. Miles, John A. Sayer, Gabrielle Wheway, Jane S. Lucas

**Affiliations:** ^1^ Translational and Clinical Research Institute, Faculty of Medical Sciences Newcastle University Newcastle upon Tyne UK; ^2^ Primary Ciliary Dyskinesia Centre, NIHR Southampton Biomedical Research Centre University of Southampton Faculty of Medicine and University Hospital Southampton NHS Foundation Trust Southampton UK; ^3^ Department of Respiratory Medicine University Hospitals NHS Trust Portsmouth UK; ^4^ Human Development and Health, Faculty of Medicine University of Southampton Southampton UK; ^5^ Wessex Regional Genetics Laboratory Salisbury NSF Foundation Trust, Salisbury District Hospital Salisbury UK; ^6^ North East Innovation Lab The Newcastle upon Tyne Hospitals NHS Foundation Trust, The Biosphere Newcastle upon Tyne UK; ^7^ Biosciences Institute, Faculty of Medical Sciences Newcastle University Newcastle upon Tyne UK; ^8^ Population Health Sciences Institute, Faculty of Medical Sciences Newcastle University Newcastle upon Tyne UK; ^9^ Renal Services Centre The Newcastle upon Tyne Hospitals NHS Foundation Trust Newcastle upon Tyne UK; ^10^ National Institute for Health Research Newcastle Biomedical Research Centre Newcastle upon Tyne UK

**Keywords:** CEP164, ciliopathy, genetics, mutation, primary ciliary dyskinesia

## Abstract

Ciliopathies may be classed as primary or motile depending on the underlying ciliary defect and are usually considered distinct clinical entities. Primary ciliopathies are associated with multisystem syndromes typically affecting the brain, kidney, and eye, as well as other organ systems such as the liver, skeleton, auditory system, and metabolism. Motile ciliopathies are a heterogenous group of disorders with defects in specialised motile ciliated tissues found within the lung, brain, and reproductive system, and are associated with primary ciliary dyskinesia, bronchiectasis, infertility and rarely hydrocephalus. Primary and motile cilia share defined core ultra‐structures with an overlapping proteome, and human disease phenotypes can reflect both primary and motile ciliopathies. *CEP164* encodes a centrosomal distal appendage protein vital for primary ciliogenesis. Human *CEP164* mutations are typically described in patients with nephronophthisis‐related primary ciliopathies but have also been implicated in motile ciliary dysfunction. Here we describe a patient with an atypical motile ciliopathy phenotype and biallelic *CEP164* variants. This work provides further evidence that *CEP164* mutations can contribute to both primary and motile ciliopathy syndromes, supporting their functional and clinical overlap, and informs the investigation and management of *CEP164* ciliopathy patients.

## Graphical abstract

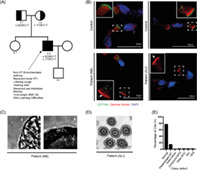

## 1 INTRODUCTION

The primary cilium is a microtubule‐based cellular appendage, which functions as a vital signalling hub with key roles in organogenesis, growth, tissue function and regeneration. It is a non‐motile organelle present on nearly every mammalian cell, consisting of a core basal body (BB), distal and sub‐distal appendages, the central ciliary axoneme, and transition zone.^1^ Primary ciliopathies are defined as a group of inherited multi‐organ diseases sharing a critical defect in the biogenesis, functioning or maintenance of the primary cilium. They display a wide spectrum of overlapping clinical manifestations such as cystic kidney disease, retinal degeneration, neurological disorders, skeletal defects, and developmental defects.

Another subtype of cilia are motile cilia, which are located as groups of organelles and are required to move fluid or debris across the cell or move the cell within fluid.^2^ They are found on specialised tissues including the respiratory tract, oviduct/fallopian tract, and ventricles of the central nervous system; the sperm flagellum is also a specialised motile cilium, as well as the embryonic nodal cilia. Motile cilia share a similar ultrastructure to primary cilia but typically have a central inner microtubule pair with accessory structures that form the motility apparatus. Abnormalities in the motile cilium typically lead to primary ciliary dyskinesia (PCD) or reduced generation of multiple motile cilia. Patients often present with defective mucociliary clearance and airway disease, including a daily wet cough from infancy, bronchiectasis, serous otitis media and rhino sinusitis. Aberrations in motile cilia can also cause male and female subfertility/infertility (common), *situs inversus* (50%), and hydrocephalus (rare).^3^

Centrosomal protein 164 kDa (CEP164) (OMIM: 614848) is a centriolar protein, localised to the distal appendages of the ciliary BB.^4^ CEP164 is essential for ciliary vesicle recruitment, correct BB docking and axonemal extension.^4^, ^5^ Recent studies have indicated that CEP164 might also be involved in motile ciliogenesis, specifically in vesicle recruitment.^6^ Biallelic *CEP164* variants have been identified in patients with nephronophthisis (NPHP)‐related ciliopathies (NPHP‐RC), a primary ciliopathy disease spectrum which can be accompanied by retinal, neurological, skeletal, and developmental abnormalities (Table S1).^7^, ^8^, ^9^, ^10^, ^11^ There are a few patients recorded with *CEP164*‐ciliopathy disease lacking a renal phenotype. More recently, *CEP164* variants have been associated with motile‐ciliary phenotypes.^12^

Here we describe a patient with bronchiectasis in whom we identified biallelic *CEP164* mutations as the likely cause of an atypical motile ciliary phenotype. This finding has important consequences for the investigation, management, and prognosis of patients with *CEP164* mutations, and for the diagnostic work up of those with PCD.

## 2 MATERIALS AND METHODS

Genetic, clinical, and in vitro investigations were carried out on a patient diagnosed with non‐cystic fibrosis (CF) bronchiectasis. Detailed methodology and ethical approval are presented within supporting information.

## 3 RESULTS

### 3.1 A patient with bronchiectasis and motile ciliary defects is explained by biallelic *CEP164* variants

**FIGURE 1:**
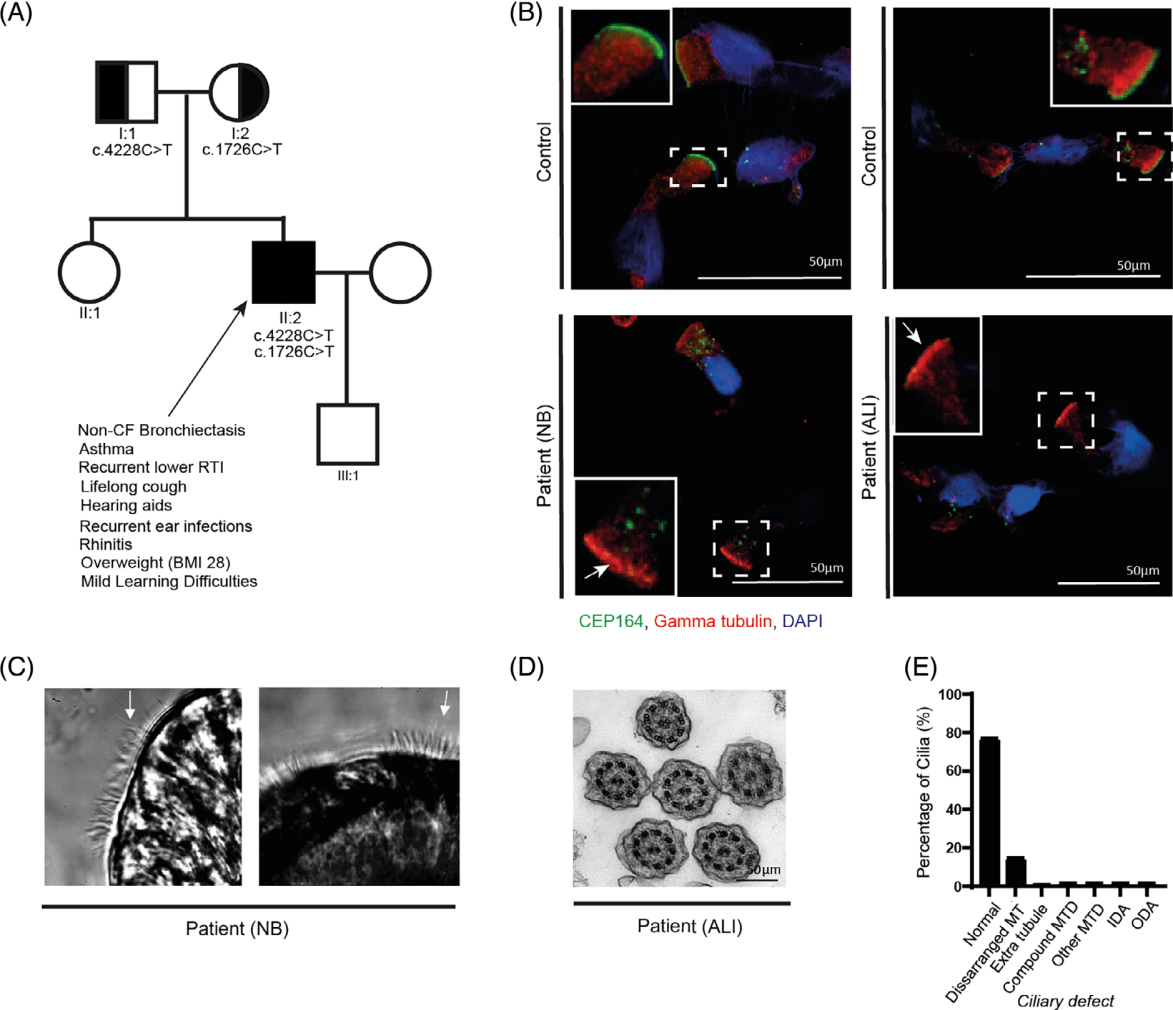
Non‐CF bronciectasis patient with *CEP164* compound heterozygous variants, motile ciliary defects and loss of CEP164 from the ciliary brush border. (A) Pedigree diagram showing the single affected male patient (arrow) with non‐CF bronchiectasis. Compound heterozygous stop gain *CEP164* variants, segregating from each parent were identified. The patient had phenotypes shown. RTI, respiratory tract infection. (B) Immunofluorescence labelling of NB and ALI cultured epithelial cells, showing expression of the centriolar marker gamma tubulin (red), CEP164 (green) and DAPI nuclear counterstain. In the healthy controls, CEP164 colocalises with gamma tubulin, marking the centriolar region. The patient's NB and ALI‐cultured cells show loss of CEP164 in the centriolar region (arrows), but some aggregate CEP164 staining remains in the cell cytoplasm. (C) Still images from high‐speed video microscopy showing the patient's NB epithelium which had uncoordinated cilia with reduced ciliary beat amplitude, and lack of mucociliary clearance. There is variable cilia length, with some long cilia (arrows). (D) TEM of patient's ALI cells show that motile cilia have a mostly normal microtubule structure. (E) Analysis of TEM images of motile cilia show that only a few cilia have microtubule defects, mainly disarranged microtubules (MT), but the ultrastructure is largely normal. The 108 cilia were counted. MT, microtubule; MTD, microtubular defect; IDA, inner dynein arm defect; ODA, outer dynein arm defect. [Colour figure can be viewed at wileyonlinelibrary.com]

The Genomics England 100,000 Genomes Project database is a rich data source for the study of ciliopathies. Filtering for biallelic or compound heterozygous *CEP164* variants in the rare disease cohort, we identified an adult patient recruited under “non‐CF bronchiectasis” in whom we identified compound heterozygous stop gain variants in *CEP164* (Figure 1A, Figure S1). The first variant, (NM_014956.5 c.1726C > T; NP_055771.4:p.R576*, NM_001271933.2:c.1735C > T, NP_001258862.1:p.R579*) was a known *CEP164*‐ciliopathy rare allele (Table 1, Table S1), with a gnomAD allele frequency of 0.000008^7^, ^12^ (Table 1). The second *CEP164* variant identified in this patient (NM_014956.5 c.4228C > T; Q1410*), was a stop gain in the penultimate exon, not been described previously in *CEP164*‐ciliopathy patients, with an allele frequency in gnomAD of 0.0008 (Table 1). Each parent of the individual was heterozygous for one *CEP164* variant, confirming segregation of these alleles. No tier 1 or 2 pathogenic variants were found within the patient's whole genome, or deleterious biallelic/compound heterozygous variants in bronchiectasis‐associated genes *CFTR, SCNN1B, SCNN1A, SCNN1G*, 42 OMIM primary ciliary dyskinesia genes or 301 gold standard Syscilia genes (Tables S2 and S3), and the patient was considered unsolved by Genomics England. There were heterozygous variants found in 5 ciliary genes (*DNAH6, MYO15A, DNAH2, BBS9, RTTN*), however none of these genes had a second pathogenic variant (Table S4).

**TABLE 1:** Compound heterozygous *CEP164* variants identified in a non‐CF bronchiectasis patient within The Genomics England 100,000 Genomes Project

Diagnosis	Sex	GRCh37	GRCh38	Transcript	Coding. change	Protein. change	Exon	Zygosity	GnomAD AF
Non‐CF bronchiectasis (Candidate PCD)	Male	11:117257920 (maternal)	11:117387204	CEP164: ENST00000278935.8	c.1726C > T	p.R576*	15/33	Het	7.9 E‐06
		11:117282575 (paternal)	11:117411859		c.4228C > T	p.Q1410*	32/33	Het	0.000815

The patient, in his 50's, of Caucasian origin, underwent deep phenotypic analysis as part of his diagnostic work‐up for non‐CF bronchiectasis, in particular PCD. The patient had a long‐standing history of recurrent lower respiratory tract infections and asthma, with a lifelong wet cough. The patient also reported recurrent ear infections, had tinnitus and wears hearing aids. The patient was also overweight, with a BMI of 28. There was no evidence of kidney or retinal defects following a renal ultrasound and ophthalmologist review, and the patient was fertile, having fathered a son. The patient had some difficulty reading and mild learning difficulties, but formal cognitive testing was not completed.

The patient was diagnosed with probable PCD. The diagnostic findings included low‐equivocal nasal nitric oxide (164 nL/min, Normal >250 nL/min, PCD usually <77 nL/min).^13^ Analysis of the patient's ciliated nasal epithelium following nasal brushings (NB) and air‐liquid‐interface (ALI) culture showed a complete loss of CEP164 protein at the cilia brush border (Figure 1B), consistent with the pathogenicity of the identified *CEP164* alleles. CEP164 intracellular aggregates were found in the NB and ALI‐cultured cells, indicating mislocalisation of the CEP164 protein. High‐speed video microscopy analysis of the NB sample showed the motile cilia had a dyskinetic‐uncoordinated ciliary beat (accurate ciliary beat frequencies was indeterminable), whilst after ALI‐culture the ciliary function appeared to have improved beat coordination with a ciliary beat frequency of 14.5 Hz (within normal ‘local’ range, at 37°C), but an abnormal ‘staggered beat’ was present. Elongated cilia were observed in both the NB and the ALI‐cultured epithelial cells; most pronounced in NB samples (Figure 1C) (Movie S1, S2, S3). Transmission electron microscopy (TEM) following ALI culture showed the motile cilia had a largely normal ultrastructure (76.9%), with only few cilia with microtubule or dynein defects (23.1%) (Figure 1D, E).

## 4 DISCUSSION

In this study we utilised the Genomics England 100,000 Genomes Project to identify and deep phenotype a non‐CF bronchiectasis patient with compound heterozygous variants in *CEP164*. One variant (NM_014956.5 c.1726C > T; R576*) was a known allele, previously found homozygous in *CEP164* patients with NPHP‐RC and motile ciliary defects.^7^, ^12^ The second variant (c.4228C > T; Q1410*) is a stop gain within the penultimate exon, not previously described in *CEP164*‐ciliopathy patients, however mutations in the final exon of *CEP164* have been previously identified.^7^ Although this second variant is annotated with ‘conflicting interpretation of pathogenicity’ in ClinVar and has a relatively high gnomAD frequency (0.0008, no homozygotes recorded), the patient's NB and ALI‐cultured epithelial cells demonstrated loss of CEP164 at the base of the motile cilia brush border, supporting the pathogenicity of the identified *CEP164* variants. CEP164 aggregates were found in the cytoplasm of the patient's cells, suggesting that CEP164 may be mislocalised, potentially due to the loss of a functioning C‐terminus which normally localises CEP164 to the centriole.^4^ The patient's NB and ALI‐cultured epithelial cells demonstrated defects in ciliary movement but ultrastructure remained largely normal. A loss of cilia was not apparent, but cilia length was occasionally elongated. We suggest that CEP164 may have roles within motile cilia aside from initial ciliary formation. This is the first *CEP164* patient described with a largely exclusive motile ciliary disease. The patient also showed a high BMI, which may further link *CEP164* variants to an obesity phenotype, as seen in ciliopathies such as Bardet‐Biedl syndrome.^8^

The findings from our patient suggest that pathogenic variants in *CEP164* can cause both primary and motile ciliopathies, correlating with known CEP164 expression patterns in the human and mouse.^14^ Indeed, there is growing evidence for ‘primary cilia genes’ to have overlapping clinical manifestations with motile ciliopathies. Some patients with pathogenic variants in *INVS* have NPHP, *situs inversus totalis*, and motile cilia dysgenesis.^15^ Also, mutations in *RPGR* and *OFD1* are found in some patients with PCD, often associated with elongated airway cilia.^16^, ^17^ Patients with polycystic kidney disease also have an increased risk of bronchiectasis, and often show PCD phenotypes.^18^ Our patient with mutations in *CEP164* was previously found in a cohort analysis of bronchiectasis patients, whereby monogenic ciliopathy gene variants were found in 12% of the cohort.^19^ With an overlapping ultrastructure, and evidence pointing towards a partially shared proteome, the role of typically ‘primary cilia’ genes in motile ciliopathies is now important. Extending genetic panels for patients with motile ciliary phenotypes such as bronchiectasis would allow more patients with these phenotypes to be diagnosed.

In conclusion, variants in *CEP164* now need to be correlated with both primary ciliopathy and motile ciliopathy phenotypes. Patients with *CEP164* genetic variants must be fully assessed and investigated for all relevant phenotypes to allow optimal and personalised management.

## AUTHOR CONTRIBUTIONS

Gabrielle Wheway, John A. Sayer, Colin G. Miles and Jane S. Lucas conceived the study with input from Laura A. Devlin. Gabrielle Wheway, Laura A. Devlin and Miguel Barroso‐Gil carried out genomic research analysis, and N. Simon Thomas carried out clinical variant analysis. Ian J. Wilson, Eric Olinger, Heather J. Cordell and Ruxandra Neatu assisted with variant interpretation. Simon A. Rock, Laura Powell and Elisa Molinari assisted in drafting and revising the manuscript. Ben Green and Jane S. Lucas clinically phenotyped the patient. Janice Coles, Claire L. Jackson, James Thompson, Woolf T. Walker, and Jane S. Lucas collected and analysed the airway data, which was interpreted with Laura A. Devlin. Laura A. Devlin wrote the initial manuscript.

## CONFLICT OF INTEREST

The authors declare no competing interests.

## Supporting information


**Appendix S1** Supporting Information

**Movie S1** Non‐CF bronchiectasis patient NB high‐speed video microscopy. Cilia move abnormally, with no coordination and no ciliary clearance. Occasional long cilia are present.

**Movie S2** Non‐CF bronchiectasis patient NB high‐speed video microscopy, movie 2, separate area to movie 1. Cilia move abnormally, no coordination and no ciliary clearance. Occasional long cilia are present.

**Movie S3** Non‐CF bronchiectasis patient ALI‐culture high‐speed video microscopy. Cilia move abnormally, but with improved beat amplitude and coordination, yet there is a ‘staggered beat’ pattern. Mucociliary clearance is seen. Ciliary beat frequency is in normal range at 37°C (14.49 Hz).

## Data Availability

Data availability statement within manuscript: “Data availability The identified variants are available within the Genomics England 100,000 Genomes project rare disease arm.”
