# Supplementary material for: Biallelic variants in CEP164 cause a motile ciliopathy‐like syndrome
Source: Clin Genet. 2022 Nov 3;103(3):330–4. doi: 10.1111/cge.14251 (PMC10099168; doi:10.1111/cge.14251)
Supplement: Supplementary file 1 — Appendix S1 Supporting Information [file CGE-103-330-s002.docx]

**Supporting Information**

**1. Acknowledgements**

Thanks to Professor Ciaran Morrison for kindly providing the monoclonal CEP164 antibody.

L.A.D is funded by the Medical Research Council Discovery Medicine North Doctoral Training Partnership (DiMeN DTP), and The Northern Counties Kidney Research Fund (NCKRF) (2019/01). J.A.S is funded by Kidney Research UK (ST_001_20171120) and the Northern Counties Kidney Research Fund. M.B-G is funded by Kidney Research UK (ST_001_20171120) and the NCKRF (2019/01). L.P is funded by the Medical Research Council Discovery Medicine North Doctoral Training Partnership. E.O is supported by the Swiss National Science Foundation (P2ZHP3_195181 & P500PB_206851) and Kidney Research UK (Paed_RP_001_20180925).

The National Primary Ciliary Dyskinesia (PCD) Centre in Southampton is commissioned and funded by NHS England; PCD research is supported by NIHR Southampton Biomedical Research Centre, NIHR Clinical Research Facility, National Institute for Health Research (RfPB PB-PG-1215-20014; and 200470) and The AAIR Charity (Reg. No. 1129698).

This research was made possible through access to the data and findings generated by the 100,000 Genomes Project. The 100,000 Genomes Project is managed by Genomics England Limited (a wholly owned company of the Department of Health and Social Care). The 100,000 Genomes Project is funded by the National Institute for Health Research and NHS England. The Wellcome Trust, Cancer Research UK and the Medical Research Council have also funded research infrastructure. The 100,000 Genomes Project uses data provided by participants and their families and collected by the National Health Service as part of their care and support.

**We acknowledge members of The Genomics England Research Consortium:**

John C. Ambrose1 ; Prabhu Arumugam1 ; Roel Bevers1 ; Marta Bleda1 ; Freya Boardman-Pretty1,2; Christopher R. Boustred1 ; Helen Brittain1 ; Mark J. Caulfield1,2; Georgia C. Chan1 ; Greg Elgar1,2; Tom Fowler1 ; Adam Giess1 ; Angela Hamblin1 ; Shirley Henderson1,2; Tim J. P. Hubbard1 ; Rob Jackson1 ; Louise J. Jones1,2; Dalia Kasperaviciute1,2; Melis Kayikci1 ; Athanasios Kousathanas1 ; Lea Lahnstein1 ; Sarah E. A. Leigh1 ; Ivonne U. S. Leong1 ; Javier F. Lopez1 ; Fiona Maleady-Crowe1 ; Meriel McEntagart1 ; Federico Minneci1 ; Loukas Moutsianas1,2; Michael Mueller1,2; Nirupa Murugaesu1 ; Anna C. Need1,2; Peter O’Donovan1 ; Chris A. Odhams1 ; Christine Patch1,2; Mariana Buongermino Pereira1 ; Daniel Perez-Gil1 ; John Pullinger1 ; Tahrima Rahim1 ; Augusto Rendon1 ; Tim Rogers1 ; Kevin Savage1 ; Kushmita Sawant1 ; Richard H. Scott1 ; Afshan Siddiq1 ; Alexander Sieghart1 ; Samuel C. Smith1 ; Alona Sosinsky1,2; Alexander Stuckey1 ; Mélanie Tanguy1 ; Ana Lisa Taylor Tavares1 ; Ellen R. A. Thomas1,2; Simon R. Thompson1 ; Arianna Tucci1,2; Matthew J. Welland1 ; Eleanor Williams1 ; Katarzyna Witkowska1,2; Suzanne M. Wood1,2.

1. Genomics England, London, UK; 2. William Harvey Research Institute, Queen Mary University of London, London, EC1M 6BQ, UK

**2. Materials and Methods**

**2.1 Ethical Statement**

This research was conducted with data and findings from the 100,000 Genomes Project. All participants in the 100K Genomes Project (100kG) provided written consent to access their anonymized clinical and genomic data for research purposes. The collection of clinical data and PCD diagnostic data for with informed consent (National Research Ethics Service South Central (A) Committee 07/Q1702/109 and University of Southampton Faculty of Medicine Ethics Committee ERGO#53155).

**2.2 The Genomics England 100,000 Genomes Project analysis**

The Genomics England 100,000 Genomes Project, is a consortium of genomic sequences from NHS rare disease or cancer patients, to enhance genetic diagnosis, and better healthcare research [1]. All participants of the 100,000 Genomes Project have provided written consent to access their anonymized genetic and clinical data for research purposes. The Genomics England 100,000 Genomes Project was approved by the Health Research Authority Research Ethics Committee East of England – Cambridge South (REC Ref 14/EE/1112) and the collection of clinical data and PCD diagnostic data for with informed consent (National Research Ethics Service South Central (A) Committee 07/Q1702/109 and University of Southampton Faculty of Medicine Ethics Committee ERGO#53155). Whole genome sequencing was performed using Illumina HiSeq 2500 sequencer, generating a mean depth of 45, and reads were aligned to the Genome Reference Consortium (GRCh38).

Two complementary methods were utilised for identification of the non-CF bronchiectasis patient described in this paper. The Newcastle research group accessed the genomic and clinical data of the rare disease cohort of patients (71,991 participants), in the main programme data release v9 (dated 2nd April 2020). Analysing tiering data within the rare disease cohort, we looked specifically for biallelic or compound heterozygous stop-gain or frameshift variants in *CEP164*. Variants from identified patients were annotated using Ensembl variant effect predictor (VEP), confirming variants in the canonical transcript; variants were selected for further analysis if potentially high impact as defined by ClinVar**.** We identified one patient who was recruited with a diagnosis of non-cystic fibrosis (CF) bronchiectasis with biallelic high impact alleles. Within the tiered variants from this individual, an analysis of putative disease-causing biallelic Tier 1 and 2 variants were completed, as well as an analysis of genes involved in primary ciliary dyskinesia (42 genes, OMIM) (Table S2) and bronchiectasis associated genes (*CFTR*, *SCNN1B*, *SCNN1A*, *SCNN1G*) [2-5]. Variants in genes known to be involved in ciliopathies were also analysed (301 Syscilia gold standard genes from Cilia Carta) [6] (Table S3).

The Southampton team analysed non-CF bronchiectasis patients tiering data for variants in known primary and motile ciliary genes, and in some other virtual panels of disease genes depending on which HPO terms were entered for a specific patient by the recruiting clinician. If variants were found in any of these applied disease gene panels, then these were tiered 1 or 2 and returned to the recruiting Genome Medicine Centre for clinical review. Our non-CF bronchiectasis patient described in this paper was recruited in Wessex GMC, and no tier 1 or tier 2 variants were returned for clinical review in this patient*.* The Southampton group analysed the patient’s variants as described in [7].

**2.3 PCD diagnostics**

We have previously described our methods for PCD diagnostics in detail [8-12]. In brief, following a focused history, nasal nitric oxide levels were measured whilst the patient exhaled against resistance to close the velum (Ecomedics CLD 88 Exhalyzer; sampling rate 0.33 l/min). Nasal brushing (NB) biopsies were taken from patients’ inferior turbinates, using a 3 mm cytology brush (Conmed, NY, USA), and the cells were analysed directly from this sample, and/or following culture at air-liquid interface.

High-speed video microscopy images of motile cilia were digitally recorded at an environmental temperature of 37 ℃ using a high-speed camera C-mounted onto an Olympus IX71 inverted light microscope and condenser; using a X100 objective lens, and recording at a rate of 500 frames per second (fps) [13]. Images were reviewed at reduced frame rates (30-60 fps) for analysis of ciliary beat pattern (CBP) and ciliary beat frequency (CBF) [9]. Ten independent and representative epithelial cell ‘clusters’ were assessed on the nasal brushings sample, and six on the ALI-culture sample after scraping with a pipette tip [8].

Transmission electron microscopy (TEM) was completed on patient culture samples. 100-300 cilia were imaged in transverse section for assessment of axonemal structure (26,500X minimum magnification for ultrastructural assessment; FEI Tecnai spirit). Quantitative and qualitative analysis were used to assess ciliary ultrastructure. Remaining basal epithelial (nasal) cells were cultured, initially in submerged culture and then on Transwell filters at an air-liquid interface for 28 days following established methodology [8]. Once fully differentiated cilia were then reanalysed to exclude secondary environmental conditions as a cause for defects in the original sample. TEM analysis was carried out according to the PCD TEM consensus standards [14].

For immunofluorescence labelling of nasal brushings and ALI cultures, cells were air dried onto slides then fixed for 15 minutes in ice cold methanol, washed in PBS and blocked for 1 hour in 1% BSA in PBS. Primary antibodies (Table S5) were incubated for 2 hours at room temperature in 1% BSA. Following PBS wash, secondary antibodies (Alexafluor 488, Life Technologies, Carlsbad, CA, USA, #A21121; Alexafluor 594, Life Technologies, Carlsbad, CA, USA, #A11012) were incubated at a dilution of 1:2500 for 30 minutes at room temperature. Cells were imaged using a Leica SP8 laser scanning confocal microscope with Leica Application Suite X software v3.5.5.19976 (Leica Biosystems, Wetzlar, Germany).


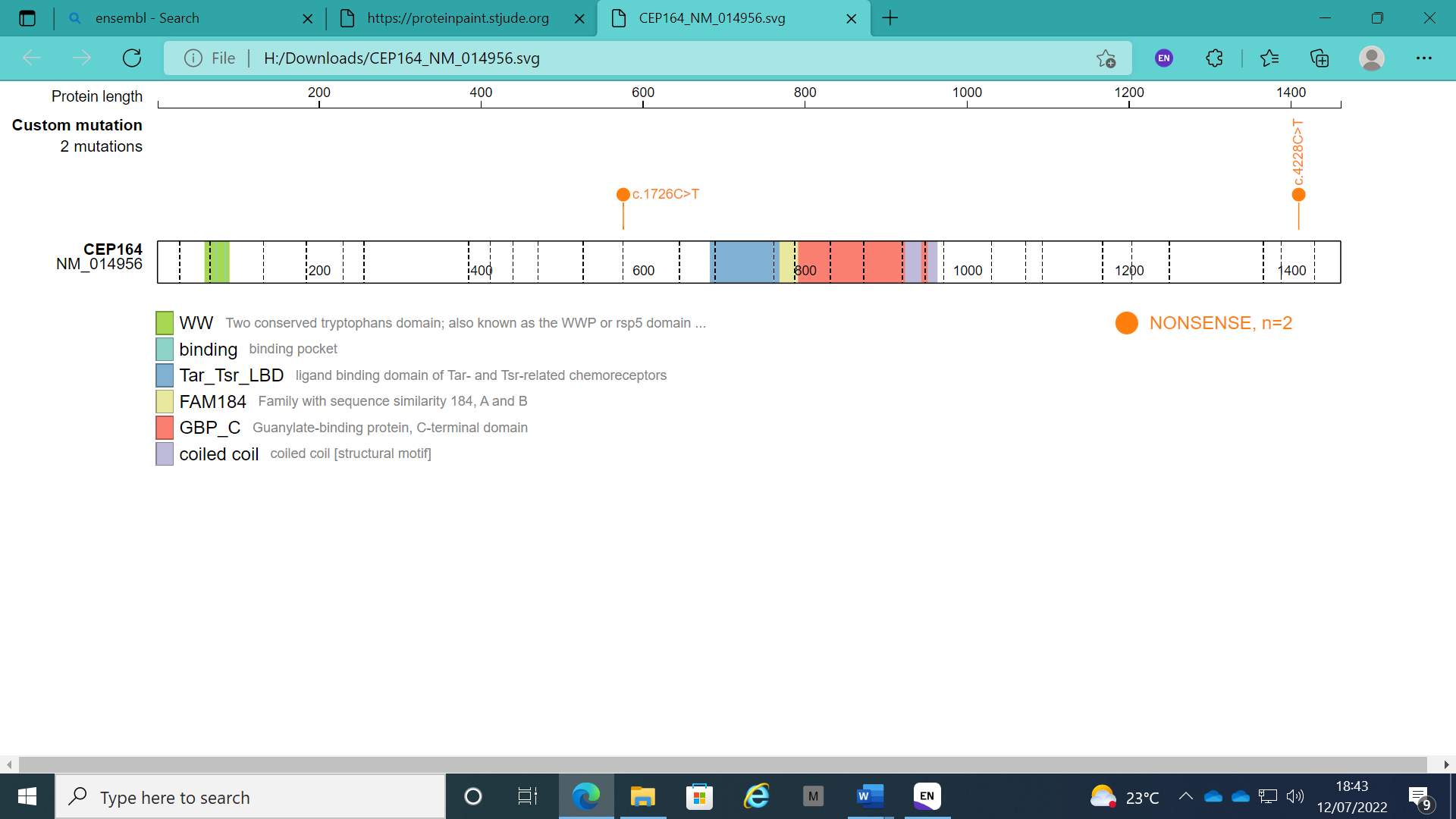
**Supporting Figures**

**Figure S1: *CEP164* variants identified in Genomics England 100,000 Genomes Project non-CF bronchiectasis patient.**

Schematic demonstrating the position of *CEP164* variants in the CEP164 protein, identified in the non-CF bronchiectasis patient. Both variants are stop gain, found in currently undefined protein regions. Schematic created using ProteinPaint ([PeCan | St. Jude Cloud (stjude.cloud)](https://pecan.stjude.cloud/proteinpaint/TP53)).

**Table S1. Description of previously identified *CEP164*-ciliopathy patients.**

| **Patients** | **Publication** | **rsID** | **GRCh37** | **Coding change** | **Protein change** | **Exon** | **Zyg** | **Kidney (ESKD)** | **Retina** | **Neurological** | **Obesity** | **PCD-like** | **Other** |
| --- | --- | --- | --- | --- | --- | --- | --- | --- | --- | --- | --- | --- | --- |
| 1, 2 | [15] | Rs387907309 | 11:117209334 | c.32A>C | p.Q11P | 03/33 | HOM | 1 = NPHP (8 y/o)  2= NPHP (8 y/o) | 1 = RD by 11 y/o  2 = - |  | 1 = Obesity ?  2 = Obesity?, |  | 2= LF? |
| 3,4,5 | [15] | Rs387907310 | 11:117222588 | c.277C>T | p.R93W | 05/33 | HET | 3 = NPHP (9 y/o)  4 = NPHP (8 y/o)  5 = Normal at 23 y/o | 3 = RD by 6 y/o  4 = LCA (blind at 5 months), NY (birth)  5 = RD by 2 y/o | 5= mild ID |  |  | 4= mild AI  5 = Seizures, DD |
|  |  | Rs387907311 | 11:117252580 | c.1573C>T | p.Q525* | 13/33 | HET |  |  |  |  |  |  |
| 6 | [15] | Rs145646425 | 11:117257920 | c.1726C>T | p.R576* | 15/33 | HOM | NPHP (8 yrs) | RD, flat ERG (not blind) | CVH | Obesity | BC (1 month) | FD, bilateral PD, abnormal LFT |
| 7 (M) | [16] | Rs145646425 | 11:117257920 | c.1726C>T | p.R576* | 15/33 | HOM | NPHP (Yes) | RP | Mild ID |  | PCD, SPI, BC | Micropenis, PD, short stature, Chronic Otis media |
| 8 | [15] | Rs1565649749 | 11:117282882 | c.4383A>G | p.*1460WextX*57 | 33/33 | HOM | Normal | LCA, flat ERG (blind by 2 yrs) |  |  |  |  |
| 9 (M), 10 (F) | [17] | Rs387907310 * | 11:117222588 | c.277C>T | p.R93W | 05/33 | HOM | 9, 10 = Normal | 9 = CRD/RP  10 = CRD/RP | 9 = ID, CA  10 = Depression and psychosis. | 9 = Obesity (BMI=33.8)  10 = Obesity (BMI = 32.9) |  | 9= HG,Gy  10 = HG, irregular menstruation. |
| 11 | [18] |  | 11:117279732 | c.3737G>A | p.R1246Q | 29/33 | HET | Adult onset NPHP |  |  |  |  |  |
|  |  |  | 11:117232609 | c.452G>A | p.R151Q | 06/33 | HET |  |  |  |  |  |  |
| 12 (F) | [19] |  | 11:117263761 | c.2535_2536dupGG | p.E846G*10 | 20/33 | HET | Symmetric hydronephrosis – no cysts |  | ID, Brain cysts, abnormal EGG, Macrocephaly, asymmetric ventriculomegaly, hypothlamic hamartoma, |  |  | OFD, PD, SD, DD, FD, down slanting palebral fissures, short stature, MD, Hypotonia, Hypertelorism, Cleft tongue, Hamartomas, absent vagina, |
|  |  | Rs746453731 | 11:117266404 | c.3055 C>T | p.Q1019* | 19/33 | HET |  |  |  |  |  |  |
|  |  |  | 470bp duplication 5q35.2 (175,668,563 – 176,138,247) |  |  |  | HET |  |  |  |  |  |  |
| 13 | [20] |  | 11:117233120 | c.195-2A>T | Upstream exon 5,199nt |  | HET | NPHP | RD | ID |  |  | LiD |
|  |  | Rs387907310 | 11:117222588 | c.277C>T | p.R93W | 05/33 | HET |  |  |  |  |  |  |

There are 13 *CEP164*-ciliopathy patients present in the literature, demonstrating large genotype-phenotype heterogeneity. Rows highlighted in grey is the known variant found in the non-CF bronchiectasis patient as described in the literature. * There is a heterozygous *BBS12* mutation (c.2014G>A), however this did not segregate in the family. Aortic insufficiency (AI), bronchiectasis (BC), body mass index (BMI), cone rod dystrophy (CRD), cerebellar aplasia (CA), cerebellar vermis hypoplasia (CVH), developmental delay (DD), electroretinogram (ERG), end-stage kidney disease (ERKD), facial dysmorphism (FD), gynecomastia (Gy), heterozygous (HET), hypogonadism (HG), homozygous (HOM), intellectual disability (ID), knockdown (KD), knockout (KO), Leber congenital amaurosis (LCA), liver disease (LiD), liver fibrosis (LF), liver function test (LFT), motor delay (MD), magnetic resonance imaging (MRI), not applicable (N/A), nephronophthisis (NPHP), nystagmus (NY), oral-facial-digital syndrome (OFD), primary ciliary dyskinesia (PCD), polydactyly (PD), retinal degeneration (RD), retinitis pigmentosa (RP), sinopulmonary infections (SPI), and Zygosity (Zyg).

**Table S2. OMIM PCD causative gene list used for variant analysis (**<https://omim.org/phenotypicSeries/PS244400>)**.**

| **Gene name** | **Ensembl ID** |
| --- | --- |
| *DNAI1* | ENSG00000122735 |
| *DNAH11* | ENSG00000105877 |
| *DNAH5* | ENSG00000039139 |
| *DNAAF3* | ENSG00000167646 |
| *HYDIN* | ENSG00000157423 |
| *NME8* | ENSG00000086288 |
| *DNAI2* | ENSG00000171595 |
| *DNAAF2* | ENSG00000165506 |
| *RSPH4A* | ENSG00000111834 |
| *RSPH9* | ENSG00000172426 |
| *DNAAF1* | ENSG00000154099 |
| *CCDC39* | ENSG00000145075 |
| *CCDC40* | ENSG00000141519 |
| *DNAL1* | ENSG00000119661 |
| *CCDC103* | ENSG00000167131 |
| *DNAAF5* | ENSG00000164818 |
| *LRRC6* | ENSG00000129295 |
| *CCDC114* | ENSG00000105479 |
| *DRC1* | ENSG00000157856 |
| *ZMYND10* | ENSG00000004838 |
| *ARMC4* | ENSG00000169126 |
| *RSPH1* | ENSG00000160188 |
| *DNAAF4* | ENSG00000256061 |
| *C21orf59* | ENSG00000265590 |
| *CCDC65* | ENSG00000139537 |
| *SPAG1* | ENSG00000104450 |
| *CCNO* | ENSG00000152669 |
| *CCDC151* | ENSG00000198003 |
| *RSPH3* | ENSG00000130363 |
| *TTC12* | ENSG00000149292 |
| *GAS8* | ENSG00000141013 |
| *DNAJB13* | ENSG00000187726 |
| *TTC25* | ENSG00000204815 |
| *PIH1D3* | ENSG00000080572 |
| *DNAH1* | ENSG00000114841 |
| *CFAP300* | ENSG00000137691 |
| *LRRC56* | ENSG00000161328 |
| *DNAH9* | ENSG00000007174 |
| *GAS2L2* | ENSG00000270765 |
| *MCIDAS* | ENSG00000234602 |
| *FOXJ1* | ENSG00000129654 |
| *NEK10* | ENSG00000163491 |

**Table S3: Syscilia gold-standard gene list from CiliaCarta, used for variant analysis [6].**

| **Gene name** | **Ensembl Gene ID** | **Gene name** | **Ensembl Gene ID** | **Gene name** | **Ensembl Gene ID** |
| --- | --- | --- | --- | --- | --- |
| *ADCY3* | ENSG00000138031 | *CEP250* | ENSG00000126001 | *EXOC3* | ENSG00000180104 |
| *AHI1* | ENSG00000135541 | *CEP290* | ENSG00000198707 | *EXOC4* | ENSG00000131558 |
| *AK7* | ENSG00000140057 | *CEP41* | ENSG00000106477 | *EXOC5* | ENSG00000070367 |
| *AK8* | ENSG00000165695 | *CEP72* | ENSG00000112877 | *EXOC6* | ENSG00000138190 |
| *ALMS1* | ENSG00000116127 | *CEP89* | ENSG00000121289 | *EXOC6B* | ENSG00000144036 |
| *ARF4* | ENSG00000168374 | *CEP97* | ENSG00000182504 | *FAM161A* | ENSG00000170264 |
| *ARL13B* | ENSG00000169379 | *CLDN2* | ENSG00000165376 | *FBF1* | ENSG00000188878 |
| *ARL3* | ENSG00000138175 | *CLUAP1* | ENSG00000103351 | *FLNA* | ENSG00000196924 |
| *ARL6* | ENSG00000113966 | *CNGA2* | ENSG00000183862 | *FOPNL* | ENSG00000133393 |
| *ASAP1* | ENSG00000153317 | *CNGA4* | ENSG00000132259 | *FOXJ1* | ENSG00000129654 |
| *ATXN10* | ENSG00000130638 | *CNGB1* | ENSG00000070729 | *FUZ* | ENSG00000010361 |
| *AZI1* | ENSG00000141577 | *CRB3* | ENSG00000130545 | *GAS8* | ENSG00000141013 |
| *B9D1* | ENSG00000108641 | *CROCC* | ENSG00000058453 | *GLI1* | ENSG00000111087 |
| *B9D2* | ENSG00000123810 | *CTNNB1* | ENSG00000168036 | *GLI2* | ENSG00000074047 |
| *BBS1* | ENSG00000174483 | *DCDC2* | ENSG00000146038 | *GLI3* | ENSG00000106571 |
| *BBS10* | ENSG00000179941 | *DFNB31* | ENSG00000095397 | *GLIS2* | ENSG00000126603 |
| *BBS12* | ENSG00000181004 | *DISC1* | ENSG00000162946 | *GPR161* | ENSG00000143147 |
| *BBS2* | ENSG00000125124 | *DNAAF1* | ENSG00000154099 | *GPR98* | ENSG00000164199 |
| *BBS4* | ENSG00000140463 | *DNAAF2* | ENSG00000165506 | *GSK3B* | ENSG00000082701 |
| *BBS5* | ENSG00000163093 | *DNAAF3* | ENSG00000167646 | *HAP1* | ENSG00000173805 |
| *BBS7* | ENSG00000138686 | *DNAH1* | ENSG00000114841 | *HEATR2* | ENSG00000164818 |
| *BBS9* | ENSG00000122507 | *DNAH10* | ENSG00000197653 | *HNF1B* | ENSG00000108753 |
| *C21orf2* | ENSG00000160226 | *DNAH11* | ENSG00000105877 | *HSPA8* | ENSG00000109971 |
| *C2CD3* | ENSG00000168014 | *DNAH2* | ENSG00000183914 | *HSPB11* | ENSG00000081870 |
| *C2orf71* | ENSG00000179270 | *DNAH5* | ENSG00000039139 | *HTR6* | ENSG00000158748 |
| *C8orf37* | ENSG00000156172 | *DNAH6* | ENSG00000115423 | *HTT* | ENSG00000197386 |
| *CBY1* | ENSG00000100211 | *DNAI1* | ENSG00000122735 | *HYDIN* | ENSG00000157423 |
| *CC2D2A* | ENSG00000048342 | *DNAI2* | ENSG00000171595 | *HYLS1* | ENSG00000198331 |
| *CCDC103* | ENSG00000167131 | *DNAL1* | ENSG00000119661 | *IFT122* | ENSG00000163913 |
| *CCDC114* | ENSG00000105479 | *DNALI1* | ENSG00000163879 | *IFT140* | ENSG00000187535 |
| *CCDC164* | ENSG00000157856 | *DPCD* | ENSG00000166171 | *IFT172* | ENSG00000138002 |
| *CCDC28B* | ENSG00000160050 | *DPYSL2* | ENSG00000092964 | *IFT20* | ENSG00000109083 |
| *CCDC37* | ENSG00000163885 | *DRD1* | ENSG00000184845 | *IFT27* | ENSG00000100360 |
| *CCDC39* | ENSG00000145075 | *DRD2* | ENSG00000149295 | *IFT43* | ENSG00000119650 |
| *CCDC40* | ENSG00000141519 | *DRD5* | ENSG00000169676 | *IFT46* | ENSG00000118096 |
| *CCDC41* | ENSG00000173588 | *DVL1* | ENSG00000107404 | *IFT52* | ENSG00000101052 |
| *CCP110* | ENSG00000103540 | *DYNC2H1* | ENSG00000187240 | *IFT57* | ENSG00000114446 |
| *CDH23* | ENSG00000107736 | *DYNLT1* | ENSG00000146425 | *IFT74* | ENSG00000096872 |
| *CENPJ* | ENSG00000151849 | *DYX1C1* | ENSG00000256061 | *IFT80* | ENSG00000068885 |
| *CEP104* | ENSG00000116198 | *EFHC1* | ENSG00000096093 | *IFT81* | ENSG00000122970 |
| *CEP135* | ENSG00000174799 | *EVC* | ENSG00000072840 | *IFT88* | ENSG00000032742 |
| *CEP164* | ENSG00000110274 | *EVC2* | ENSG00000173040 | *INPP5E* | ENSG00000148384 |
| **Gene name** | **Ensembl Gene ID** | **Gene name** | **Ensembl Gene ID** | **Gene name** | **Ensembl Gene ID** |
| *HTR6* | ENSG00000158748 | *NEK4* | ENSG00000114904 | *RABL5* | ENSG00000128581 |
| *HTT* | ENSG00000197386 | *NEK8* | ENSG00000160602 | *RAN* | ENSG00000132341 |
| *HYDIN* | ENSG00000157423 | *NGFR* | ENSG00000064300 | *RANBP1* | ENSG00000099901 |
| *HYLS1* | ENSG00000198331 | *NIN* | ENSG00000100503 | *RFX3* | ENSG00000080298 |
| *IFT122* | ENSG00000163913 | *NINL* | ENSG00000101004 | *RILPL1* | ENSG00000188026 |
| *IFT140* | ENSG00000187535 | *NME5* | ENSG00000112981 | *RILPL2* | ENSG00000150977 |
| *IFT172* | ENSG00000138002 | *NME7* | ENSG00000143156 | *ROPN1L* | ENSG00000145491 |
| *IFT20* | ENSG00000109083 | *NME8* | ENSG00000086288 | *RP1* | ENSG00000104237 |
| *IFT27* | ENSG00000100360 | *NOTO* | ENSG00000214513 | *RP2* | ENSG00000102218 |
| *IFT43* | ENSG00000119650 | *NPHP1* | ENSG00000144061 | *RPGR* | ENSG00000156313 |
| *IFT46* | ENSG00000118096 | *NPHP3* | ENSG00000113971 | *RPGRIP1* | ENSG00000092200 |
| *IFT52* | ENSG00000101052 | *NPHP4* | ENSG00000131697 | *RPGRIP1L* | ENSG00000103494 |
| *IFT57* | ENSG00000114446 | *NUP214* | ENSG00000126883 | *RSPH1* | ENSG00000160188 |
| *IFT74* | ENSG00000096872 | *NUP35* | ENSG00000163002 | *RSPH3* | ENSG00000130363 |
| *IFT80* | ENSG00000068885 | *NUP37* | ENSG00000075188 | *RSPH4A* | ENSG00000111834 |
| *IFT81* | ENSG00000122970 | *NUP62* | ENSG00000213024 | *RSPH9* | ENSG00000172426 |
| *IFT88* | ENSG00000032742 | *NUP93* | ENSG00000102900 | *RTTN* | ENSG00000176225 |
| *INPP5E* | ENSG00000148384 | *OCRL* | ENSG00000122126 | *SASS6* | ENSG00000156876 |
| *INTU* | ENSG00000164066 | *ODF2* | ENSG00000136811 | *SCLT1* | ENSG00000151466 |
| *INVS* | ENSG00000119509 | *OFD1* | ENSG00000046651 | *SDCCAG8* | ENSG00000054282 |
| *IQCB1* | ENSG00000173226 | *ORC1* | ENSG00000085840 | *Sep-02* | ENSG00000168385 |
| *KIF17* | ENSG00000117245 | *PACRG* | ENSG00000112530 | *Sep-07* | ENSG00000122545 |
| *KIF19* | ENSG00000196169 | *PAFAH1B1* | ENSG00000007168 | *SGK196* | ENSG00000185900 |
| *KIF24* | ENSG00000186638 | *PARD3* | ENSG00000148498 | *SHH* | ENSG00000164690 |
| *KIF27* | ENSG00000165115 | *PARD6A* | ENSG00000102981 | *SLC47A2* | ENSG00000180638 |
| *KIF3A* | ENSG00000131437 | *PCDH15* | ENSG00000150275 | *SMO* | ENSG00000128602 |
| *KIF3B* | ENSG00000101350 | *PCM1* | ENSG00000078674 | *SNAP25* | ENSG00000132639 |
| *KIF3C* | ENSG00000084731 | *PDE6D* | ENSG00000156973 | *SNX10* | ENSG00000086300 |
| *KIF7* | ENSG00000166813 | *PDZD7* | ENSG00000186862 | *SPA17* | ENSG00000064199 |
| *LCA5* | ENSG00000135338 | *PHF17* | ENSG00000077684 | *SPAG16* | ENSG00000144451 |
| *LRRC6* | ENSG00000129295 | *PIBF1* | ENSG00000083535 | *SPAG17* | ENSG00000155761 |
| *LZTFL1* | ENSG00000163818 | *PKD1* | ENSG00000008710 | *SPAG6* | ENSG00000077327 |
| *MAK* | ENSG00000111837 | *PKD1L1* | ENSG00000158683 | *SPATA7* | ENSG00000042317 |
| *MAL* | ENSG00000172005 | *PKD2* | ENSG00000118762 | *SPEF2* | ENSG00000152582 |
| *MAPRE1* | ENSG00000101367 | *PKHD1* | ENSG00000170927 | *SSNA1* | ENSG00000176101 |
| *MCHR1* | ENSG00000128285 | *PLK1* | ENSG00000166851 | *SSTR3* | ENSG00000183473 |
| *MDM1* | ENSG00000111554 | *POC1A* | ENSG00000164087 | *STIL* | ENSG00000123473 |
| *MKKS* | ENSG00000125863 | *PTCH1* | ENSG00000185920 | *STK36* | ENSG00000163482 |
| *MKS1* | ENSG00000011143 | *PTPDC1* | ENSG00000158079 | *STK38L* | ENSG00000211455 |
| *MLF1* | ENSG00000178053 | *RAB11A* | ENSG00000103769 | *STOML3* | ENSG00000133115 |
| *MNS1* | ENSG00000138587 | *RAB11FIP3* | ENSG00000090565 | *STX3* | ENSG00000166900 |
| *MYO15A* | ENSG00000091536 | *RAB17* | ENSG00000124839 | *SUFU* | ENSG00000107882 |
| *MYO7A* | ENSG00000137474 | *RAB23* | ENSG00000112210 | *SYNE2* | ENSG00000054654 |
| *NEK1* | ENSG00000137601 | *RAB3IP* | ENSG00000127328 | *TBC1D30* | ENSG00000111490 |
| *NEK2* | ENSG00000117650 | *RAB8A* | ENSG00000167461 | *TBC1D7* | ENSG00000145979 |
| **Gene name** | **Ensembl Gene ID** | **Gene name** | **Ensembl Gene ID** |  |  |
| *TCTN1* | ENSG00000204852 | *TULP3* | ENSG00000078246 |  |  |
| *TCTN2* | ENSG00000168778 | *ULK4* | ENSG00000168038 |  |  |
| *TCTN3* | ENSG00000119977 | *USH1C* | ENSG00000006611 |  |  |
| *TEKT2* | ENSG00000092850 | *USH1G* | ENSG00000182040 |  |  |
| *TEKT4* | ENSG00000163060 | *USH2A* | ENSG00000042781 |  |  |
| *TEKT5* | ENSG00000153060 | *VDAC3* | ENSG00000078668 |  |  |
| *TMEM138* | ENSG00000149483 | *VHL* | ENSG00000134086 |  |  |
| *TMEM216* | ENSG00000187049 | *WDPCP* | ENSG00000143951 |  |  |
| *TMEM231* | ENSG00000205084 | *WDR19* | ENSG00000157796 |  |  |
| *TMEM237* | ENSG00000155755 | *WDR35* | ENSG00000118965 |  |  |
| *TMEM67* | ENSG00000164953 | *WDR60* | ENSG00000126870 |  |  |
| *TNPO1* | ENSG00000083312 | *WDR78* | ENSG00000152763 |  |  |
| *TOPORS* | ENSG00000197579 | *XPNPEP3* | ENSG00000196236 |  |  |
| *TPPP2* | ENSG00000179636 | *ZNF423* | ENSG00000102935 |  |  |
| *TRAF3IP1* | ENSG00000204104 |  |  |  |  |
| *TRAPPC10* | ENSG00000160218 |  |  |  |  |
| *TRAPPC3* | ENSG00000054116 |  |  |  |  |
| *TRAPPC9* | ENSG00000167632 |  |  |  |  |
| *TRIM32* | ENSG00000119401 |  |  |  |  |
| *TRIP11* | ENSG00000100815 |  |  |  |  |
| *TTBK2* | ENSG00000128881 |  |  |  |  |
| *TTC12* | ENSG00000149292 |  |  |  |  |
| *TTC21B* | ENSG00000123607 |  |  |  |  |
| *TTC26* | ENSG00000105948 |  |  |  |  |
| *TTC29* | ENSG00000137473 |  |  |  |  |
| *TTC30A* | ENSG00000197557 |  |  |  |  |
| *TTC30B* | ENSG00000196659 |  |  |  |  |
| *TTC8* | ENSG00000165533 |  |  |  |  |
| *TTK* | ENSG00000112742 |  |  |  |  |
| *TTLL3* | ENSG00000214021 |  |  |  |  |
| *TTLL6* | ENSG00000170703 |  |  |  |  |
| *TTLL9* | ENSG00000131044 |  |  |  |  |
| *TUBA1A* | ENSG00000167552 |  |  |  |  |
| *TUBA1C* | ENSG00000167553 |  |  |  |  |
| *TUBA4A* | ENSG00000127824 |  |  |  |  |
| *TUBB2A* | ENSG00000137267 |  |  |  |  |
| *TUBB2B* | ENSG00000137285 |  |  |  |  |
| *TUBB3* | ENSG00000198211 |  |  |  |  |
| *TUBE1* | ENSG00000074935 |  |  |  |  |
| *TUBGCP2* | ENSG00000130640 |  |  |  |  |
| *TUBGCP3* | ENSG00000126216 |  |  |  |  |
| *TUBGCP4* | ENSG00000137822 |  |  |  |  |
| *TUBGCP5* | ENSG00000153575 |  |  |  |  |
| *TUBGCP6* | ENSG00000128159 |  |  |  |  |
| *TULP1* | ENSG00000112041 |  |  |  |  |

**Table S4: Heterozygous variants in ciliary genes identified in the Genomics England 100,000 Genomes project non-CF bronchiectasis patient.**

| **Gene** | **GRCh38** | **Zygosity** | **Consequence** | **Transcript** | **Coding.**  **change** | **Protein.**  **change** | **GnomAD AF** | **Mutation_**  **taster** |
| --- | --- | --- | --- | --- | --- | --- | --- | --- |
| *DNAH6* | 2:84713177 | Het | Missense | ENST000000389384.8 | c.9461C>T | p.L3154P | N/A | Disease causing |
| *MYO15A* | 17:18142796 | Het | Missense | ENST000000647156.2 | c.5866C>T | p.R1956W | 2.693E-04 | Disease causing |
| *DNAH2* | 17:7780194 | Het | Missense | ENST000000572933.5 | c.5760A>C | p.K1920N | 3.979E-06 | Disease causing |
| *BBS9* | 7:33273825 | Het | Spice Acceptor | ENST000000242067.1 | c.887-2A>G | Splice defect | N/A |  |
| *RTTN* | 18:70166983 | Het | Missense | ENST000000640769.2 | c.1738C>T | p.R5880C | 8.916E-08 | Polymorphism |

**Table S5: Primary antibodies used for immunofluorescence staining.**

| Primary IgG | Manufacturer | Dilution |
| --- | --- | --- |
| Anti-CEP164 (Monoclonal) | Made by Professor Ciaran Morrison | 1:200 |
| Anti-gamma tubulin | Sigma-Aldrich (T5326) | 1:500 |

**Movie S1**: **Non-CF bronchiectasis patient NB high-speed video microscopy.** Cilia move abnormally, with no coordination and no ciliary clearance. Occasional long cilia are present.

**Movie S2**: **Non-CF bronchiectasis patient NB high-speed video microscopy, movie 2, separate area to movie 1**. Cilia move abnormally, no coordination and no ciliary clearance. Occasional long cilia are present.

**Movie S3: Non-CF bronchiectasis patient ALI-culture high-speed video microscopy.** Cilia move abnormally, but with improved beat amplitude and coordination, yet there is a ‘staggered beat’ pattern. Mucociliary clearance is seen. Ciliary beat frequency is in normal range at 37°C (14.49 Hz).

**References**

1. Turnbull, C., et al., *The 100 000 Genomes Project: bringing whole genome sequencing to the NHS.* BMJ, 2018. **361**: p. k1687.

2. Casals, T., et al., *Bronchiectasis in adult patients: an expression of heterozygosity for CFTR gene mutations?* Clin Genet, 2004. **65**(6): p. 490-5.

3. Sheridan, M.B., et al., *Mutations in the beta-subunit of the epithelial Na+ channel in patients with a cystic fibrosis-like syndrome.* Hum Mol Genet, 2005. **14**(22): p. 3493-8.

4. Azad, A.K., et al., *Mutations in the amiloride-sensitive epithelial sodium channel in patients with cystic fibrosis-like disease.* Hum Mutat, 2009. **30**(7): p. 1093-103.

5. Mutesa, L., et al., *Genetic analysis of Rwandan patients with cystic fibrosis-like symptoms: identification of novel cystic fibrosis transmembrane conductance regulator and epithelial sodium channel gene variants.* Chest, 2009. **135**(5): p. 1233-1242.

6. van Dam, T.J.P., et al., *CiliaCarta: An integrated and validated compendium of ciliary genes.* PLoS One, 2019. **14**(5): p. e0216705.

7. Wheway, G., et al., *Whole genome sequencing in the diagnosis of primary ciliary dyskinesia.* BMC Med Genomics, 2021. **14**(1): p. 234.

8. Coles, J.L., et al., *A Revised Protocol for Culture of Airway Epithelial Cells as a Diagnostic Tool for Primary Ciliary Dyskinesia.* Journal of Clinical Medicine, 2020. **9**(11).

9. Rubbo, B., et al., *Accuracy of High-Speed Video Analysis to Diagnose Primary Ciliary Dyskinesia.* Chest, 2019. **155**(5): p. 1008-1017.

10. Shoemark, A., et al., *International consensus guideline for reporting transmission electron microscopy results in the diagnosis of primary ciliary dyskinesia (BEAT PCD TEM Criteria).* Eur Respir J, 2020. **55**(4).

11. Shoemark, A., et al., *Topological data analysis reveals genotype-phenotype relationships in primary ciliary dyskinesia.* Eur Respir J, 2021. **58**(2).

12. Jackson, C.L., et al., *Accuracy of diagnostic testing in primary ciliary dyskinesia.* Eur Respir J, 2016. **47**(3): p. 837-48.

13. Hirst, R.A., et al., *Culture of Primary Ciliary Dyskinesia Epithelial Cells at Air-Liquid Interface Can Alter Ciliary Phenotype but Remains a Robust and Informative Diagnostic Aid.* Plos One, 2014. **9**(2).

14. Shoemark, A., et al., *International consensus guideline for reporting transmission electron microscopy results in the diagnosis of primary ciliary dyskinesia (BEAT PCD TEM Criteria).* European Respiratory Journal, 2020. **55**(4).

15. Chaki, M., et al., *Exome capture reveals ZNF423 and CEP164 mutations, linking renal ciliopathies to DNA damage response signaling.* Cell, 2012. **150**(3): p. 533-48.

16. Shamseldin, H.E., et al., *The morbid genome of ciliopathies: an update.* Genet Med, 2020. **22**(6): p. 1051-1060.

17. Maria, M., et al., *Genetic and clinical characterization of Pakistani families with Bardet-Biedl syndrome extends the genetic and phenotypic spectrum.* Sci Rep, 2016. **6**: p. 34764.

18. Fujimaru, T., *Genetic Background and Clinicopathologic Features of Adult-onset nephronophthisis* 2021.

19. Strong, A., et al., *Expanding the genetic landscape of oral-facial-digital syndrome with two novel genes.* Am J Med Genet A, 2021. **185**(8): p. 2409-2416.

20. Vilboux, T., et al., *Molecular genetic findings and clinical correlations in 100 patients with Joubert syndrome and related disorders prospectively evaluated at a single center.* Genet Med, 2017. **19**(8): p. 875-882.
